# The testobolome in microbial testosterone metabolism and human health

**DOI:** 10.1038/s41522-025-00861-0

**Published:** 2026-01-09

**Authors:** Liisa Veerus, Anushka Subrahmanian, Martin J. Blaser

**Affiliations:** 1 Center for Advanced Biotechnology and Medicine, Rutgers University, Piscataway, NJ USA; 2 Robert Wood Johnson Medical School, Rutgers University, New Brunswick, NJ USA

**Keywords:** Computational biology and bioinformatics, Microbiology

## Abstract

We propose the term testobolome, analogous to the estrobolome, to describe gut bacteria that metabolize testosterone. Testosterone undergoes microbial transformations similar to estrogens, potentially influencing host hormone homeostasis and health. This review defines the testobolome, identifies its known members, and explores mechanisms that are shared or distinct from the estrobolome. We outline a framework for future research into microbiome-mediated steroid metabolism, including its role in aging and hormone-driven diseases.

## Entering the era of microbiome-sex hormone interactions

**Fig. 1:**
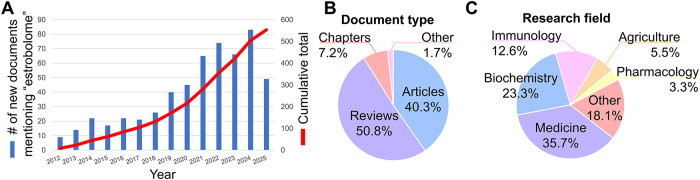
Analysis of research documents that mention the term “estrobolome” between 2011–2025 (*n* = 553). Data from Elsevier’s Scopus citation database (accessed on August 14, 2025) indicates that: (**A**) interest in the estrobolome has increased in recent years, (**B**) with the majority of documents being reviews (**C**) predominantly in research fields related to human medicine. Interest in the estrobolome across diverse fields underscores the timeliness of considering the testobolome and encourages original research to complement the growing body of reviews.

**Fig. 2:**
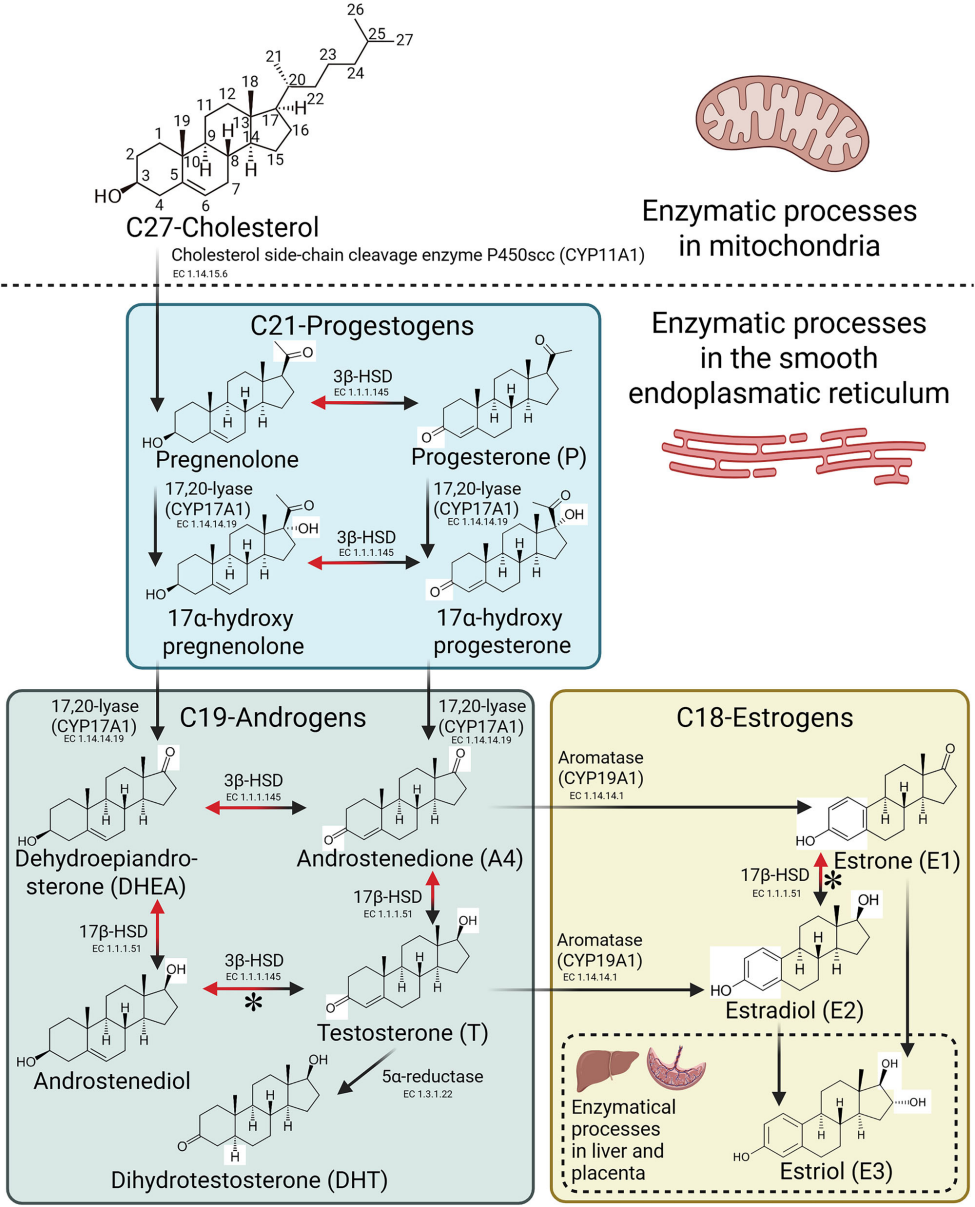
Biosynthesis of C19-androgens and C18-estrogens from C27-cholesterol^162^. Cholesterol is enzymatically cleaved in mitochondria, giving rise to C21-progestogens that are precursors for both androgens and estrogens. All sex steroids are synthesized in the smooth endoplasmic reticulum of tissue-specific steroidogenic cells. In the ovary, theca cells convert cholesterol to androgens, which are aromatized to estrone and estradiol in adjacent granulosa cells^18^. Estriol is primarily produced in the liver or placenta during pregnancy via the hydroxylation of estrone and estradiol^163,164^. In the testis, Leydig cells synthesize testosterone as the primary androgen^165^. In men, estrogens are produced peripherally via the aromatization of circulating testosterone and androstenedione in adipose tissue, brain, bone, and to a lesser extent in testes. The figure indicates the specific modifications in the chemical structures of the steroids in comparison to their immediate precursors (white rectangles), and the steroid biosynthetic enzymes with their enzyme commission (EC) numbers. Although steroidogenesis overwhelmingly favors forward reactions due to the rapid use of downstream products, some conversions may be reversible (red arrows), with those most likely to occur indicated (*). The remaining conversions are thermodynamically and structurally irreversible. 3β-HSD – 3-beta-hydroxysteroid dehydrogenase, 17,20-lyase – steroid 17-alpha-hydroxylase, 17β-HSD – 17-beta-hydroxysteroid dehydrogenase. Figure created with BioRender.

In 2011, the term “estrobolome” was introduced, defining it as a collection of enteric bacteria and their gene products that metabolize estrogens^1^. By mid-2025, the paper by Plottel and Blaser proposing the concept had been cited >550 times according to Scopus and >760 according to Google Scholar. With the increasing interest in estrogen-metabolizing bacteria (Fig. 1), and their role in health and disease, in this review, we reflect on advancements made and lessons learned in the estrobolome field during the past 15 years. We posit that an orthologous phenomenon relates to testosterone, the main sex hormone in males, which could also be metabolized by bacteria, i.e., the “testobolome”^2^. While the broader term “androbolome” has been used^3–5^, we believe it is specifically testosterone and its microbial metabolism that warrant focused attention, given testosterone’s status as the most abundant circulating androgen, its role as a central precursor to both estrogens and dihydrotestosterone (DHT; Fig. 2), and its disproportionate potential to impact health.

## Sex steroid biosynthesis

Sex steroids underlie reproductive system maturation, secondary sex characteristics, and behavioral development in female and male vertebrate animals^6–8^, with related compounds with differential biosynthesis and functions detected in some invertebrates^9^ and plants^10^. Their production diverges in sexually mature individuals, resulting in estrogens primarily in females and androgens, particularly testosterone, in males.

In humans and other mammals, the C18-estrogen hormone family includes three major endogenous sex hormones that are derived from C27-cholesterol (Fig. 2): estradiol (E2), which is synthesized in the ovaries from C21-progestogens through androstenedione and testosterone intermediates, and is abundant in pre-menopausal women during their reproductive years; estriol (E3), which is upregulated in pregnancy; and estrone (E1), which is synthesized in the adipose tissue^11,12^, and is the dominant estrogen in post-menopausal women^13^.

Testosterone is also synthesized downstream of C27-cholesterol and C21-progestogens, but upstream of estrogen biosynthesis (Fig. 2), making testosterone together with androstenedione precursors to all estrogen family members^14^. In males, testosterone synthesis declines with age after the end of reproductive peak at around age 50, but this transition is less abrupt than that for estrogens in menopause^15^.

## Regulation of sex hormones

The hypothalamic-pituitary-gonadal (HPG) axis orchestrates the synthesis of both estrogens and androgens^16^. In females, the hypothalamus secretes gonadotropin-releasing hormone (GnRH) in pulses, stimulating the anterior pituitary to release luteinizing hormone (LH) and follicle-stimulating hormone (FSH)^17^. These gonadotropins act on the ovaries—LH on theca cells and FSH on granulosa cells—to promote estrogen production^18^. Estrogens feed back to the hypothalamus and pituitary depending on their concentration and menstrual cycle stage^19^.

**Fig. 3:**
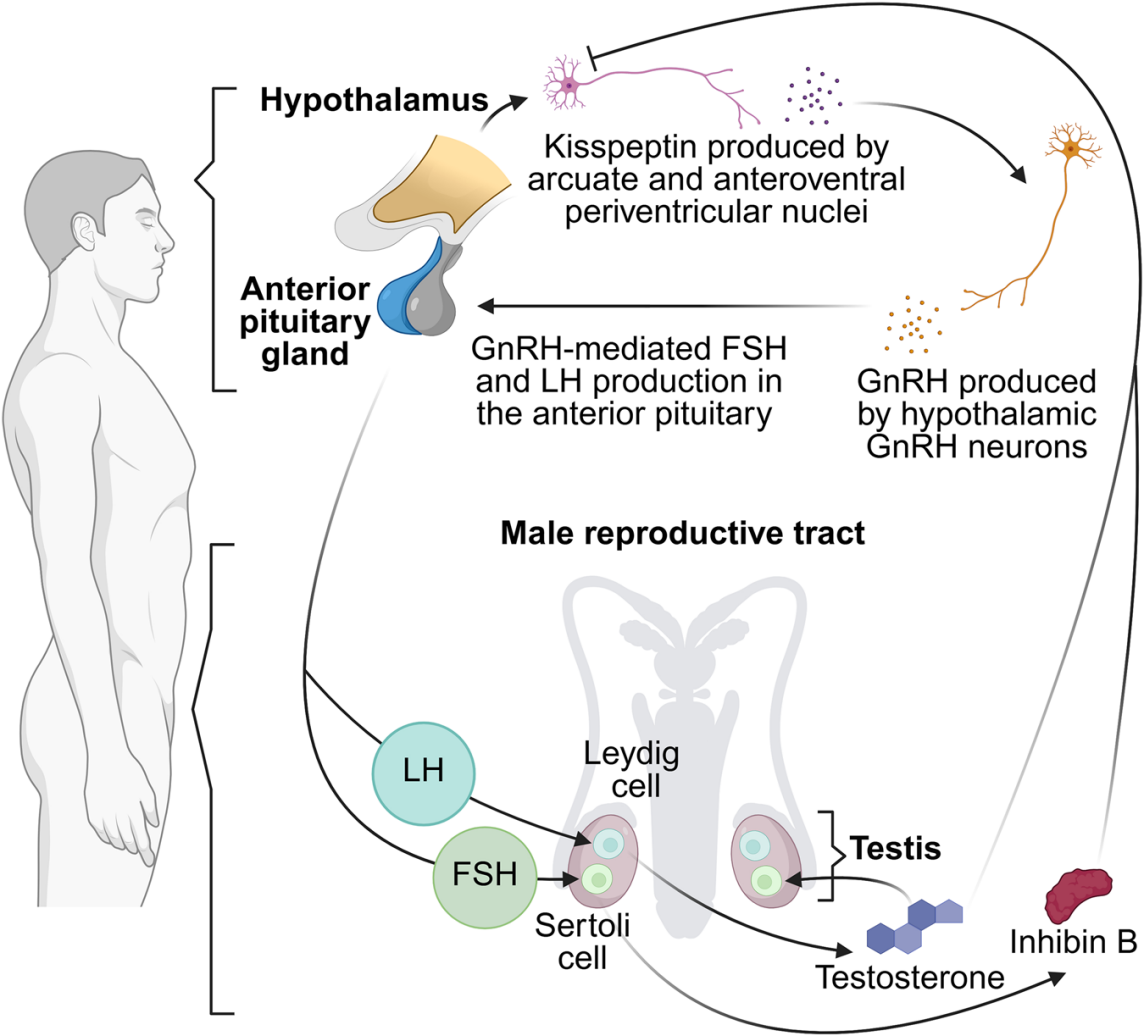
Hierarchical regulation of testosterone synthesis in the male hypothalamic-pituitary-gonadal (HPG) axis. Specialized neurons initiate the axis by releasing kisspeptin, which binds to KISS1R on gonadotropin-releasing hormone (GnRH) neurons^166,167^. This stimulates pulsatile GnRH secretion, which triggers the anterior pituitary to produce luteinizing hormone (LH) and follicle-stimulating hormone (FSH) that promote steroidogenesis in the gonads. LH stimulates Leydig cells in the testes to produce testosterone through a multi-step steroidogenic process: cholesterol is imported into mitochondria via steroidogenic acute regulatory protein (StAR) and converted to pregnenolone by cholesterol desmolase CYP11A1^168^. Subsequent enzymatic steps, primarily via the CYP17A1, 3β-HSD, and 17β-HSD enzymes (Fig. 2), yield testosterone, which is secreted into circulation and also acts locally within the testes to support spermatogenesis. Figure created with BioRender.

Similarly, in males, GnRH-induced release of LH and FSH from the anterior pituitary targets the testes, where LH specifically binds to receptors on Leydig cells to stimulate testosterone synthesis and FSH acts on Sertoli cells to support spermatogenesis and maintain the testicular environment in conjunction with testosterone (Fig. 3)^20,21^. Rising testosterone levels provide negative feedback to the hypothalamus and pituitary, ensuring homeostasis within the HPG axis^22^. Inhibin B, produced by Sertoli cells, selectively downregulates FSH, providing an additional layer of regulation within the male HPG axis. Since LH and FSH are regulated by separate feedback loops—testosterone and inhibin B, respectively—testosterone production and spermatogenesis can be functionally decoupled, allowing for more precise modulation of male reproductive function^21,23,24^.

In both sexes, the lower-abundance sex steroids, such as estrogens in males and androgens in females, are also regulated by the same HPG axis and additionally synthesized in extra-gonadal tissues, including the adrenal glands, bone, brain, skin, and adipose tissue^25^. It remains poorly understood whether and how host-associated bacteria may participate in the regulation axis and impact the sex steroid pools^26^.

## Endocrine trajectories over the lifespan

**Fig. 4:**
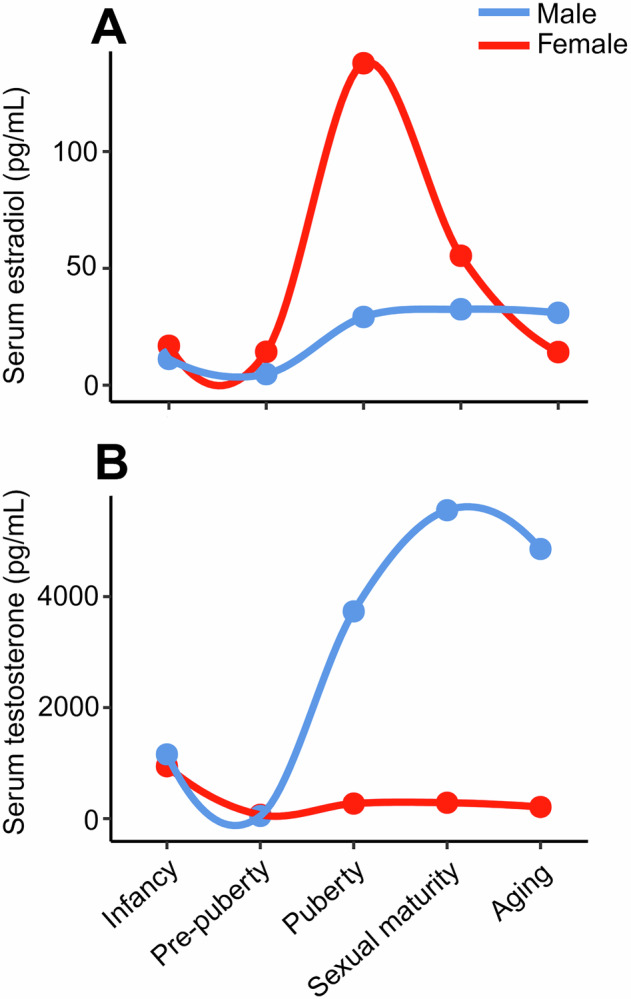
Lifespan trajectories of primary sex hormones in humans. Schematic of mean serum (**A**) estradiol and (**B**) testosterone concentrations across five major life stages: infancy/early childhood (0–5 years), pre-puberty (6–11 years), puberty/adolescence (12–17 years), sexual maturity [reproductive years] (18–50 years), and post-menopause/aging (51–80 years). This figure was generated de novo by the authors by extracting and averaging reported values from multiple independent studies^169–178^ that met the following inclusion criteria: healthy human participants, no use of hormone-modulating medications, and quantitation of total circulating sex steroid levels in serum using mass spectrometry. The elevated sex steroid levels in early childhood cause a “mini-puberty” that kickstarts sex steroid synthesis^28,29^. Importantly, circulating hormone levels do not completely reflect their activity in tissues, where many cells can locally produce or convert sex steroids for their own use (intracrine) or for nearby cells (paracrine)^179^.

Circulating levels of estradiol and testosterone exhibit distinct sex-specific trajectories across the lifespan (Fig. 4). In females, estradiol is low in early life, surges at puberty, fluctuates cyclically during reproductive years, and drops sharply after menopause. In males, estradiol remains low and stable, produced via peripheral aromatization of testosterone. Notably, postmenopausal women have lower estradiol than age-matched men, reflecting the continued testosterone-to-estradiol conversion in men and the loss of ovarian production in women^27^.

Testosterone in males peaks transiently in infancy (“mini-puberty”; important in both females and males to kickstart the HPG axis^28,29^), rises sharply at puberty, remains high through adulthood, and gradually starts to decline with age (~1% annual reduction)^30^. In females, testosterone is present at infancy, increases modestly at adrenarche (adrenal glands start producing DHEA and DHEAS to prepare for puberty^31^), then remains low and stable through adulthood.

These temporally-conserved hormonal fluctuations may be shaped by gut microbes in unanticipated ways, highlighting a critical, understudied axis in endocrine biology.

## Sex steroid induced cellular signaling

Synthesized testosterone and estrogens circulate in serum, with the majority ( > 97%)^13^ being reversibly bound (inactive form) to one of two proteins synthesized by the liver: sex-hormone binding globulin (SHBG)^32^ or albumin, which binds with ~20–25% of the affinity of SHBG^33^. Protein-binding allows lipophilic steroids to be transported in the bloodstream^34^ without degradation or activation of receptors^33^.

Due to its lower affinity, albumin, the most abundant plasma protein, allows increased receptor interactions compared to SHBG and also can bind to phytoestrogens (plant estrogens) that could affect endocrine homeostasis^34^. For example, phytoestrogens (present in legumes, particularly soy) are metabolized by the gut microbiota into bioactive compounds with either estrogenic or anti-estrogenic properties^35^. These can then affect endogenous estrogen homeostasis by enhancing estrogenic activity in postmenopausal women or disrupting hormonal balance in men, and also contribute to sex-specific susceptibility to metabolic, reproductive, and hormone-dependent cancers^35^.

While sex hormone binding specificities vary, estrogens are generally bound to albumin (52–54% bound in females and 68–74% in males) and testosterone to SHBG (62% in females and 43–45% in males)^36,37^. A small fraction of testosterone may bind to circulating corticosteroid-binding globulin (CBG)^38^ or to an SHBG ortholog, androgen-binding protein (ABP), within the seminiferous tubules, where ABP aids in concentrating testosterone to the levels necessary for sustaining spermatogenesis^32,34^. SHBG increases with age in men, reducing bioavailable testosterone which contributes to andropause, the gradual decline in androgen activity despite relatively stable total testosterone levels^39^.

To exert their biological effects, estrogens and testosterone must first be dissociated from their protein carriers. Dissociation occurs because steroid-protein binding is reversible, and tissue receptors have far higher binding affinity than carrier proteins, especially albumin, resulting in an equilibrium balanced toward receptor binding^40^. The use of two carriers with different affinities (high-affinity SHBG for stability and low-affinity albumin for rapid exchange) balances long-term hormone homeostasis with the ability to respond quickly to local demand for sex steroids^41^. Estrogens have several well-characterized nuclear and membrane-bound receptors (Table 1**)**. Membrane receptors rapidly trigger transcription-independent signaling. This may occur, for example, by activating the MAPK/ERK cascade, PI3K/Akt survival signaling, Src kinase pathways, and second messenger systems such as cAMP/PKA and PLC/IP₃/DAG, which together mediate calcium mobilization, cytoskeletal remodeling via Rho GTPases, and transactivation of growth factor receptors like EGFR75^42^; this ultimately regulates cell processes including proliferation, motility, and metabolism. Testosterone binds to nuclear (AR)^43^ and membrane-specific androgen receptors (mARs)^44^. The ARs are paralogous to estrogen receptors as members of the nuclear receptor superfamily^45^, while the mARs are poorly characterised and may be structurally unrelated.

**Table 1:** Sex hormone receptors in humans and their mechanisms of action

Binding hormone	Cellular location of the receptor	Receptor name	Receptor mode of action
Estrogens	Nucleus	ERα^129^	Transcription factor
		ERβ^129^	
	Membrane	mERα^130^	Signal transduction
		mERβ^130^	
		GPER1 (GPR30)^131^	G-protein coupled receptor
		ER-X^*^^132^	
		ERx^*^^133^	
		Gq-mER^*^^134–136^	
Testosterone (and other androgens)	Nucleus	AR^43^	Transcription factor
	Membrane	TRPM8*^44^	Calcium ion channel
		OXER1*^44^	G-protein coupled receptor (inhibited by testosterone)
		GPRC6A*^44^	G-protein coupled receptor
		ZIP9*^44^	Zinc transporter

The outlined estrogen receptors bind all or a selection of estrogen family members at different affinity (estradiol > estrone > estriol)^137^. A similar gradient of binding affinities is observed for the androgen receptors (dihydrotestosterone (DHT) > testosterone > androstenedione)^138^. Those receptors that have been proposed, but not confirmed, to bind sex steroids are indicated by *.

## Conjugation and clearance of excess steroids

**Fig. 5:**
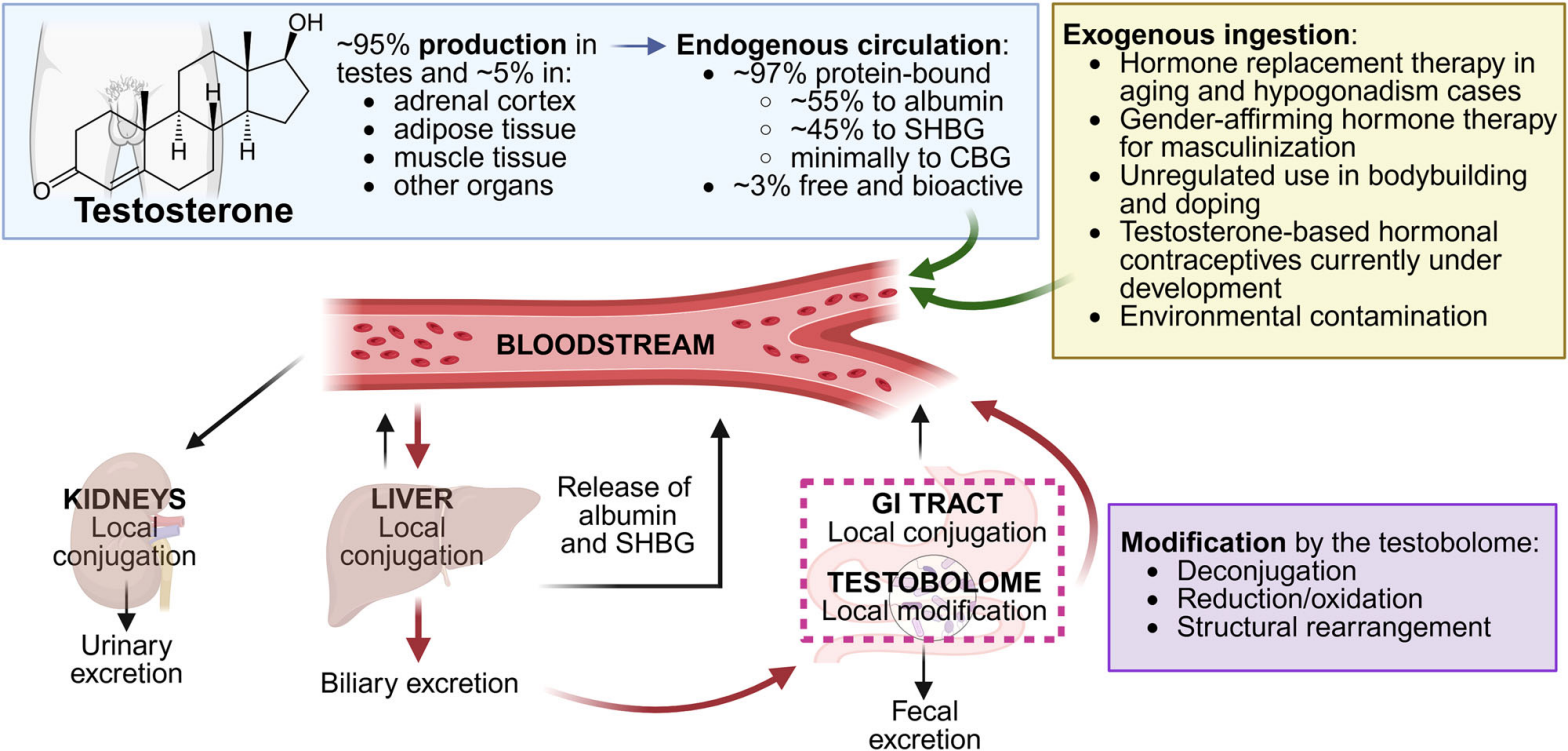
The interaction between testosterone metabolism and the intestinal microbiome (testobolome). Testosterone, whether endogenous (blue box) or exogenously acquired (yellow box), circulates systemically in the bloodstream (green arrows) and is gradually inactivated by the liver and, to a lesser extent, the kidneys, where it undergoes sequential modification: the addition or exposure of hydroxyl (–OH) or keto ( = O) groups to increase polarity (phase I metabolism), followed by conjugation with charged groups like glucuronic acid or sulfate (phase II metabolism) to increase water solubility^180^. The liver is considered the central site for testosterone metabolism and bioactivity regulation, as it not only catalyzes hormone clearance but also produces albumin and SHBG, which bind circulating testosterone and govern its systemic availability. Conjugation renders previously hydrophobic sex steroid hormone hydrophilic, facilitating the excretion of testosterone metabolites primarily via urine (after renal or hepatic processing) or secondarily via bile (hepatic processing), followed by fecal elimination. In the lumen of the gastrointestinal (GI) tract, conjugated hormones secreted in bile may undergo microbial transformation via the testobolome (pink dashed box), including deconjugation and structural modifications (purple box). These microbial processes can reactivate bioactive sex steroids otherwise destined for excretion. The resulting compounds may then be reabsorbed into the bloodstream, contributing to enterohepatic recirculation (red arrows) and potentially elevating systemic sex steroid levels. Among the organs involved, only the liver and intestines contribute meaningfully to testosterone reabsorption, while the kidneys primarily serve as an excretory route. SHBG – sex hormone-binding globulin, CBG – corticosteroid-binding globulin. Figure created with BioRender.

Sex steroids are transported via the bloodstream to the liver (Fig. 5), where their fate—either retention in the circulation or inactivation via phase II conjugation reactions^46^—is determined by their protein-binding status: bound sex steroids are protected from immediate hepatic metabolism, while unbound hormones are available for uptake into hepatocytes. Conjugation marks the steroids for excretion, either through the kidneys or through the biliary tract into the intestinal canal. In humans, the conjugation of estrogens involves glucuronidation by UDP-glucuronosyltransferases (UGTs) of the 1A and 2B families, sulfonation by sulfotransferases (SULTs; particularly the cytosolic SULT1E1), and *O*-methylation via catechol-*O*-methyltransferase (COMT)^47^. In parallel, testosterone is conjugated primarily through glucuronidation by UGT2B15 and UGT2B17, and UGT2B7 to a lesser degree^48^, with minor contributions from sulfonation by SULT2A1^49^. Conjugation also occurs in the kidneys and intestine (Fig. 5), and to some extent in adrenal tissue and lungs^50^.

Conjugation enhances the hydrophilicity of otherwise lipophilic sex hormones (Fig. 5). Due to their increased polarity, conjugated molecules rely on active transport for excretion, mediated by epithelial efflux transporters, such as members of the MRP (multidrug resistance-associated protein), BCRP (breast cancer resistance protein), and BSEP (bile salt export pump) families^51^. While the determinants of the specificity of the excretion route are not well-understood at present, urinary excretion is expected to be the major pathway due to its rapid elimination of sex steroids, while biliary excretion plays a secondary role^52,53^. This pattern may reflect physicochemical properties, as smaller, more polar and thus more hydrophilic molecules are more readily cleared by the kidneys, whereas larger, more hydrophobic conjugates are preferentially excreted into bile^46^.

Genetic polymorphisms, such as the UGT2B17 deletion^54^, significantly reduce the urinary excretion of testosterone, particularly in those with a homozygous deletion^55^; the reduced urinary testosterone excretion occurs without elevating circulating levels likely due to (undefined) negative feedback mechanisms (Fig. 3)^55^. Thus, local testosterone exposure in target tissues like the prostate may still be increased.

## Evidence of the testobolome in action

The testobolome refers to the gut microbes and their enzymes that modify testosterone and related androgens, altering their activity, promoting reabsorption, or generating novel metabolites. These transformations can influence systemic androgen levels and host physiology. Among the most compelling evidence for the testobolome comes from functional studies showing that gut microbes can modulate testosterone bioavailability, with primary support for testosterone-microbiome interdependence stemming from perturbation studies. Germ-free male mice have lower plasma testosterone levels than conventional mice that have an intact microbiome^56^. This finding suggests that intestinal microbes are part of the normal cycle of sex steroid biosynthesis. Similarly, having a microbiome or conventionalizing germ-free mice with a butyrate-producing strain (*Clostridium tyrobutyricum*, phylum Bacillota) elevated intratesticular testosterone levels and restored testicular function compared to a germ-free control group^57^. In the intestine, germ-free mice also exhibit dramatically reduced concentrations of active androgens, such as 5-androstendiol, accompanied by an accumulation of inactive glucuronide conjugates, consistent with the role of the microbiome in local steroid activation^58^. Together, these findings suggest that having a microbiome promotes sex steroid biosynthesis, HPG axis activation, and the emergence of secondary sex characteristics.

Treatment of male mice with the broad-spectrum antibiotic ciprofloxacin suppressed the expression of nuclear receptor subfamily 4 group A member 1 (NR4A1) and its downstream target, steroidogenic acute regulatory protein (StAR), thereby reducing testosterone biosynthesis, which led to lower circulating testosterone, impaired spermatogenesis, and testicular tissue damage^59^. This suggests that antibiotic exposure can disrupt endocrine regulation, although the mechanism of action, whether involving the microbiome or not, was not determined. Similarly, early-life exposure to the broad-spectrum tetracycline antibiotic, doxycycline, reduced male mouse plasma testosterone and impaired spermatogenesis, as shown by the thinning of the seminiferous epithelium, reduced sperm count, and increased LH^60^. Therefore, doxycycline may act as an endocrine-disrupting chemical (EDC) by causing mitochondrial dysfunction in Leydig cells, leading to reduced expression of key steroidogenic enzymes such as CYP11A1, CYP17A1, and 17β-HSD, and ultimately impairing testosterone biosynthesis^60^.

Considering the importance of mini-puberty (Fig. 4) in mammals for establishing long-term reproductive and metabolic programming, such findings raise concern that early-life antibiotic-induced microbiome disruption^61^ may interfere with testosterone-dependent developmental processes and possibly affect fertility later in life. However, restoration may be possible. In male mice, colistin-induced suppression of testosterone production was related to depletion of genus *Akkermansia* (phylum Verrucomicrobiota) and disrupted inosine metabolism^62^. Inosine supplementation restored testosterone levels, possibly by enhancing intestinal barrier integrity and reducing systemic lipopolysaccharide levels, factors known to influence testicular immune signaling and steroidogenic function^62^.

## Microbial genes, enzymes, and pathways modulating testosterone

The microbial genes and enzymes modulating sex steroids have been identified largely from estrobolome and bile acid metabolism research. However, many of these findings can be extrapolated to testosterone given the shared biosynthesis and elimination pathways (Figs. 2 and 5).

**Fig. 6:**
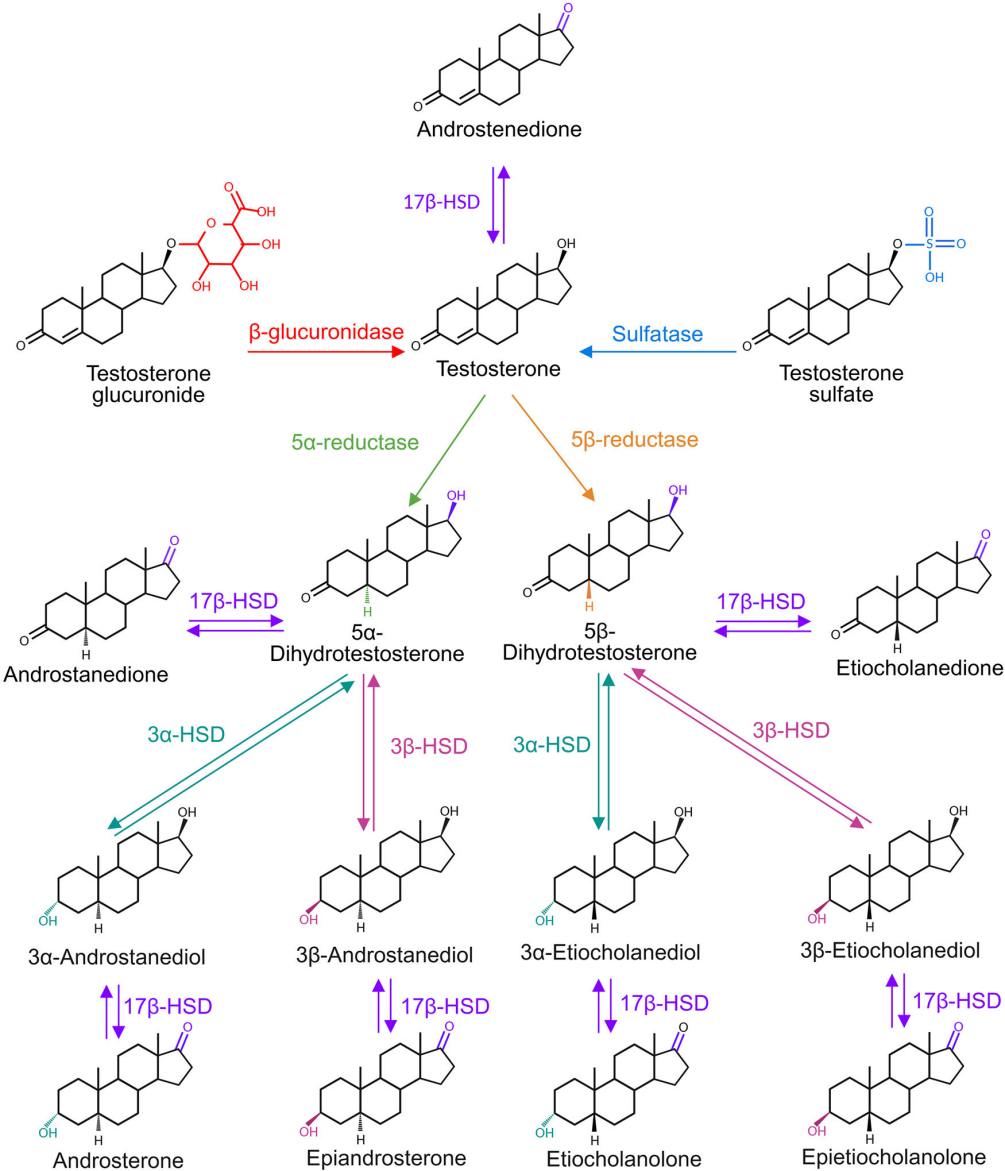
Known and proposed microbial enzymatic conversions of testosterone and its derivatives. Conjugated testosterone (testosterone glucuronide and testosterone sulfate) can be deconjugated by β-glucuronidase (red) and sulfatase (blue), generating free testosterone. Bacterial 17β-hydroxysteroid dehydrogenases (17β-HSDs; purple) catalyze the reversible interconversion between testosterone and androstenedione, as well as downstream oxidized and reduced metabolites. Reductions at the A-ring are carried out by 5α-reductase (green) and 5β-reductase (orange), forming 5α- and 5β-dihydrotestosterone (DHT) isomers, respectively. These can be further reversibly transformed into diols by 3α-HSD (teal) and 3β-HSD (pink), and ultimately into ketosteroids such as androsterone, epiandrosterone, etiocholanolone, and epietiocholanolone. The active site of chemical modification is color-coded in each structure to match the responsible enzyme. All transformations shown are bacterially catalyzed, highlighting the capacity of the gut microbiome to regulate androgen metabolism via deconjugation, redox reactions, and structural modifications. Reductive direction is normally favored under anaerobic gut conditions. Figure created with BioRender.

*Reactivation of testosterone via deconjugation*. Many intestinal bacterial species produce β-glucuronidases (Table 2), GUS enzymes encoded by *gus* or *bg* genes^63,64^, that hydrolyze glucuronic acid from glucuronide conjugates, including estrogen glucuronides and likely testosterone glucuronides (Fig. 6)^65,66^. Both male and female germ-free mice exhibit significantly higher levels of conjugated testosterone in the cecum and colon than their conventional counterparts^58,67^, providing evidence that microbial activity underlies testosterone deconjugation in the gut of both sexes. This can be attributed to the lack of GUS enzymes detected in the cecal contents of germ-free mice compared to conventionalized germ-free mice and conventional mice, suggesting that most cecal GUS is of bacterial origin^68^. In contrast, high GUS concentrations in the feces have been inversely correlated with fecal estrogen concentrations in humans^69^, implying that the presence of GUS facilitates the reactivation and reabsorption of estrogens. Gut microbial GUS can also hydrolyze testosterone-glucuronide into free testosterone, as shown by enzyme- and donor-dependent activity in both recombinant and human fecal assays^70^. Supporting this mechanism, early-life exposure to doxycycline was shown to significantly alter the gut microbiota of male mice and reduce testosterone levels, an effect that may be mediated by an increase in the genus *Ruminococcus (*phylum Bacillota*)*^60^. This genus is known to encode both GUS (Table 2) and 3β-HSD (Table 3), implicating it in both the reactivation of conjugated testosterone and its further transformation into bioactive androgens. This finding illustrates that antibiotic-induced microbiota shifts may indirectly disrupt host androgen levels by expanding taxa with steroid-modifying enzymatic capabilities.

**Table 2:** Major human gut bacterial taxa known to include strains able to produce GUS enzymes^139–141^

Phylum	Class	Family	Genus (species)
Bacillota (formerly Firmicutes)	Clostridia	Clostridiaceae	*Clostridium (perfringens)*^142^
		Ruminococcaceae	*Ruminococcus (gnavus)*^143^
		Lachnospiraceae	*Faecalibacterium (prausnitzii)*^66,143^ *Roseburia (hominis* and *intestinalis)*^66^
		Eubacteriaceae	*Eubacterium*^63^
	Bacilli	Streptococcaceae	*Streptococcus*^142^
		Lactobacillaceae	*Lactobacillus (rhamnosus)*^143^
		Enterococcaceae	*Enterococcus*^142^
		Bacillaceae	*Bacillus*^142^
		Staphylococcaceae	*Staphylococcus*^142^
Bacteroidota (formerly Bacteroidetes)	Bacteroidia	Bacteroidaceae	*Bacteroides (vulgatus* and *fragilis)*^144^
		Tannerellaceae	*Parabacteroides (merdae)*^145^
Actinomycetota (formely Actinobacteria)	Actinobacteria	Bifidobacteriaceae	*Bifidobacterium (longum, pseudolongum, bifidum, angulatum*, and *breve)*^144^
		Corynebacteriaceae	*Corynebacterium*^142^
Pseudomonadota (formerly Proteobacteria)	Gammaproteobacteria	Enterobacteriaceae	*Escherichia (coli)*^142^ *Klebsiella*^142^
		Moraxellaceae	*Acinetobacter*^142^
Acidobacteriota (formerly Acidobacteria)	Acidobacteriia	Acidobacteriaceae	*Acidobacterium (capsulatum)*^146^

Phyla are ordered from most to least abundant in the healthy human gut. Bacillota, accounting for >95% of *gus*-associated and >40% of *bg*-associated sequences in humans^147^, is the key phylum associated with β-glucuronidase activity. A distinct phylogenetic subclass of GUS enzymes has been identified in phylum Bacteroidota, suggesting functional diversity and broader phylogenetic distribution than previously recognized^140^.

**Table 3:** Microbial enzymes contributing to the testobolome and their functional roles in androgen metabolism

Microbial enzyme	Reported in bacteria	Functional consequence
3α-HSD	*Lachnolostridium scindens*^148^, *Eggerthella lenta*^149–151^, *Arthrobacter koreensis*^107^, *Parabacteroides distasonis*^151^, *Raoultibacter timonensis*^151^, *Gordonibacter pamelaeae*^151^, *Clostridium perfringens*^153^, *Comamonas testosteroni*^148^, *Streptomyces hydrogenans*^152^	↓ androgen potency; weak ERβ activation (via androstanediol)
3β-HSD	*Pseudomonas nitroreducens*^85^, *Eggerthella lenta*^149,151^, *Gordonibacter pamelaeae*^151^, *Ruminococcus gnavus*^150^, *Clostridium innocuum*^75^, *Clostridium perfringens*^153^, *Klebsiella aerogenes*^154^, *Parabacteroides merdae*^151^, *Odoribacter laneus*^151^, Odoribacteraceae spp^151^, *Mycobacterium leprae*^155^, *Comamonas testosteroni*^71,156^	↓ androgen potency; potential ERβ activation (via androstanediol)
17β-HSD	*Pseudomonas nitroreducens*^85^, *Comamonas testosteroni*^71,156^, *Rhodococcus* sp P14^157^, *Lysinibacillus sphaericus* DH-B01^158^, *Pseudomonas putida* SJTE-1^159^	Interconversion between active (17β-hydroxy) and inactive (17-keto) androgens/estrogens; can either increase or decrease potency depending on direction
5β-reductase	*Lachnolostridium scindens*^161^*,* *Clostridium innocuum*^75^, *Clostridium hylemonae*^151^, *Bacteroides* and *Parabacteroides* strains ^151^,	Androgen inactivation; enhanced clearance (via 5β-androstanes)
5α-reductase	*Parabacteroides merdae*^151^, *Odoribacter laneus*^151^, Odoribacteraceae spp^151^, *Bacteroides dorei*^151^, *Alistipes* sp^151^, Bacteroides and *Parabacteroides* strains^151^ Bacteroidetes bacteria^160^, Proteobacteria bacteria^160^, Deltaproteobacteria bacterium^160^, Spirochaetae bacterium HGW-Spirochaetae-1^160^, Saprospirales bacterium^160^, *Chitinophaga niastensis*^160^, *Chitinophaga ginsengisegetis*^160^, *Chitinophaga arvensicola*^160^, *Chitinophaga* sp GDMCC 1.1325^160^, *Sandaracinus* sp^160^	↑ androgen potency (if DHT is produced)

Enzymes are listed from top to bottom in the order of highest to lowest reported prevalence in the healthy human gut and decreasing experimental confidence in their activity within gut microbes. Human gut-associated bacteria are underlined, and listed from most to least abundant in the healthy human gut. Non-underlined taxa represent environmental species not typically reported in the normal human microbiome but potentially acquired from the surroundings.

Microbial interaction with host hormones ultimately depends on their bioavailability. Compared to estradiol, testosterone is more abundant and is subject to less fluctuation across sexes and life stages (Fig. 4), potentially making it a more consistent and accessible substrate for bacteria (Fig. 5). We hypothesize that this stability likely increases the susceptibility of testosterone to bacterial interactions and modulation. While the relationship between systemic hormone stability and microbial modulation remains to be tested, *Comamonas testosteroni* JLU460ET (phylum Pseudomonadota) provides preliminary evidence that some gut microbes may preferentially metabolize testosterone over estradiol due to its stronger transcriptional induction of steroid-degrading genes^71^.

*Reactivation of testosterone via desulfonation*. Testosterone sulfation is a quantitatively minor conjugation route compared to glucuronidation and contrasts with the abundance of sulfated estrogens^72,73^. The capacity of gut microbes to hydrolyze sulfate esters via sulfatases (Fig. 6) provides an additional mechanism for modulating host testosterone availability. Ser-type enzymes encoded by Bacteroidetes represent ~80% of gut microbial sulfatases^74^; this phylum is the primary source of testosterone desulfation, while the remaining Cys-type sulfatases found in Firmicutes, Proteobacteria and Actinobacteria also may contribute to this reactivation^74^.

*Interconversion of androgens via microbial redox reactions*. Beyond deconjugation, intestinal microbes encode reductive and oxidative enzymes (Table 3) that can directly modify the steroid structure of testosterone and related androgens by adding or removing hydrogen atoms, respectively. These redox reactions alter hormone structure, activity, and metabolic stability, influencing both potency and clearance. For example, gut bacteria may alter the double bond structure of the testosterone backbone through 5α- and 5β-reductases^75^, which catalyze irreversible reduction of the Δ4 double bond, generating stereoisomeric metabolites with distinct metabolic fates (Fig. 6).

Catalyzing reversible redox reactions at the steroid backbone C3 and C17 positions, 3α-, 3β-, and 17β-hydroxysteroid dehydrogenases (HSDs) are present in human gut microbiota (Table 3)^76^. These microbial enzymes share catalytic functions with human HSDs (Fig. 2) but differ in substrate preference, stereospecificity, and physiological role. In humans, 3β-HSDs are critical for steroid hormone endogenous biosynthesis, converting Δ5-steroids like pregnenolone and dehydroepiandrosterone (DHEA) into Δ4-ketosteroids, such as progesterone and androstenedione (Fig. 2)^77^. In contrast, microbial 3α- and 3β-HSDs more commonly act on downstream androgens, including DHT (Fig. 6), converting it into 3α- or 3β-androstanediols and other metabolites^76,77^. Importantly, 3α- and 3β-androstanediol are less potent androgens and instead exert weak estrogen-like effects, binding to ERβ^78^ in tissues, including prostate, gut, and brain (Supplementary Fig. 1), where they trigger anti-inflammatory responses^79^. With these selective activities, such microbial metabolites could become tissue-specific ERβ agonists for therapeutic use. Similarly, while human 17β-HSDs regulate the interconversion between testosterone and androstenedione (and between estradiol and estrone; Fig. 2), microbial 17β-HSDs may contribute to the reactivation or inactivation of these hormones in the gut (Fig. 6, Table 3), affecting local and systemic hormone homeostasis and availability. Currently, such activities are host-specific and unmeasured, but they could be harnessed for preventive, diagnostic, and therapeutic purposes.

*Additional microbial modulation of testosterone*. Gut microbes may mediate a range of biochemical transformations that further diversify testosterone fate. These include epimerization (inversion of stereochemistry at hydroxyl-bearing carbons), which can alter receptor binding; hydroxylation at several carbon positions, introducing new sites for phase II conjugation; and side chain cleavage, which can generate structurally simplified androgens with distinct activity profiles. Current knowledge of microbial androgen epimerization is very limited; the only example identified is epitestosterone, formed from androstenedione via 17α-HSD activity reported in *Lachnoclostridium scindens* (phylum Bacillota)^80^. This reaction differs from the typical role of microbial 17α-HSD, which carries out oxidation-reduction at the 17α position, because it inverts the orientation of the hydroxyl group at carbon 17 (β to α). This stereochemical “flip” changes the three-dimensional shape of the hormone and can markedly reduce its ability to activate the androgen receptor^81^. Microbial hydroxylation, as reported for *Bacillus megaterium* (phylum Bacillota) and *Sorangium cellulosum* (phylum Myxococcota), introduces a new hydroxyl group at a novel position on the steroid backbone, creating additional sites for conjugation and potentially altering receptor interactions^82^.

Some microbes may also perform de novo synthesis of steroid-like metabolites from host- or diet-derived precursors, potentially contributing to an expanded pool of hormonally active molecules. While less well-characterized than GUS, sulfatases, or HSD enzymes, these processes outlined below highlight the underappreciated metabolic plasticity of the gut microbiome in shaping androgenic signaling.

## Microbial cross-hormone transformations and catabolism

Certain gut-associated or environmentally enriched bacterial strains have the capacity for cross-hormone conversion or catabolic degradation of testosterone and related steroids^83^. For example, *L. scindens*, a human gut microbe at high prevalence, can convert glucocorticoids into testosterone^84^. The testosterone-degrading bacterium *Pseudomonas nitroreducens* (phylum Pseudomonadota) found in human microbiome may contribute to testosterone deficiency-associated hyperlipidemia^85^. In contrast, anaerobic estrogenesis was observed in *Phosphitispora* sp strain TUW77 (phylum Bacillota), isolated from the gut of the great blue-spotted mudskipper (*Boleophthalmus pectinirostris*), which ferments testosterone to produce estrogens and androstanediol under anaerobic conditions^86^. *C. testosteroni* can degrade testosterone for its own carbon needs^87^. Although usually considered as an opportunistic microbiome member, its metabolic activity exemplifies microbial contributions to androgen depletion.

Species from the genus *Denitratisoma* (phylum Pseudomonadota), present in anaerobic aquatic sediments, can retroconvert estrogens into androgens via cobalamin-dependent methylation^88^.

While some of these taxa are not human gut-associated, their enzymatic capacity indicates the broader potential of microbes to interconvert sex steroids. Such findings suggest that the testobolome may influence not just testosterone levels, but also the broader balance between androgenic and estrogenic signaling.

## Consequences of disordered testosterone metabolism

**Fig. 7:**
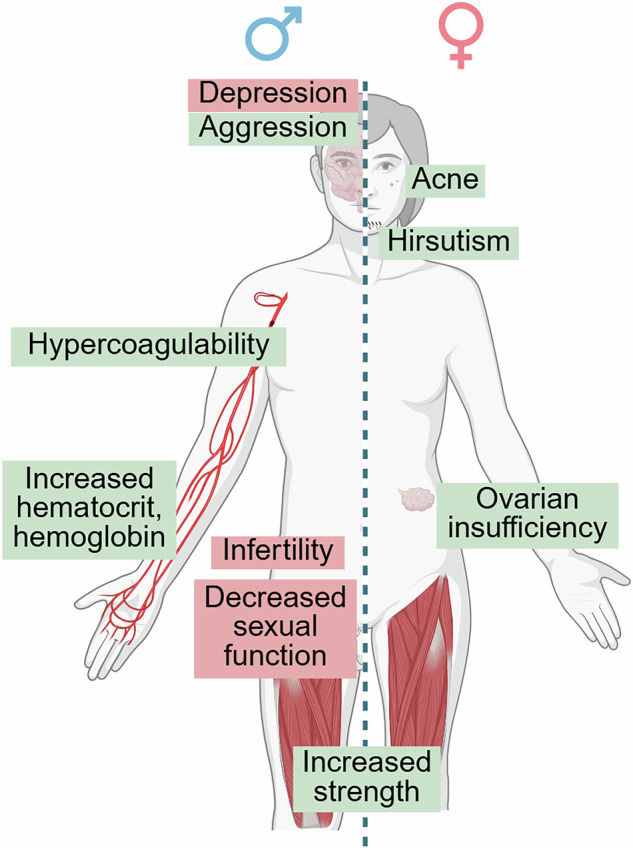
Effects of disordered testosterone metabolism. Known consequences of excess testosterone (green) and testosterone deficiency (red) are shown for both men (left) and women (right). Figure created with BioRender.

Sex steroid hormones and microbes engage in bidirectional interactions: microbes modulate host sex steroids to support their own survival, while the changed hormone landscape, in turn, influences host responses^89^. Because of this interplay, disruptions in testosterone metabolism can have effects that extend beyond endocrine regulation, contributing to a range of well-described reproductive, metabolic, and behavioral disorders in both men and women (Fig. 7).

The presence of testosterone can select for particular bacteria. Men with higher serum testosterone have more diverse gut communities, with genera such as *Acinetobacter* (phylum Pseudomonadota), *Dorea* (phylum Bacillota), *Ruminococcus* (phylum Bacillota), and *Megamonas* (phylum Bacillota) significantly increased in the feces, while women with higher estradiol have more Bacteroidota and fewer Bacillota, with some genera like *Slackia* (phylum Actinomycetota) and *Butyricimonas* (phylum Bacteroidota) inversely correlated with high serum estradiol^90^. Testosterone and its derivatives can affect virulence and antibiotic resistance in the microbiome. Castrated mice, which have lower circulating testosterone levels, show reduced bacterial load in skin abscesses and diminished production of virulence factors by inoculated *Staphylococcus aureus* (phylum Bacillota)^91^. In *Pseudomonas aeruginosa* (phylum Pseudomonadota), testosterone and DHT decreased MIC for tobramycin but increased it for cefepime, while in *Enterococcus faecalis* (phylum Bacillota) they raised MICs for meropenem and norfloxacin, respectively^92^, indicating hormone-induced shifts in resistance phenotypes.

The health impacts of disrupted testosterone metabolism and testobolome span multiple systems and are evident in both sexes. In men, hypogonadism—defined by low circulating testosterone—is associated with diminished sexual function, impaired spermatogenesis, and reduced fertility. Clinical trials have shown that administering testosterone can reverse sexual dysfunction in both healthy men undergoing hormone suppression^93^ and in men with diagnosed hypogonadism^94^. Additionally, therapeutic modulation of testosterone production, for example with clomiphene, has been shown to improve fertility outcomes in men^95^. Whether these effects are mediated via the testobolome is currently unknown.

In women, hyperandrogenism is a hallmark of polycystic ovarian syndrome (PCOS)^96^, where excess testosterone can drive hirsutism through 5α-reductase-mediated (human origin) conversion to DHT^97^, exacerbate acne severity^98^, and potentially impair ovarian function via inflammation^99^ or altered follicular morphology^100^. Elevated levels of bacterial β-glucuronidase and β-glucosidase have been proposed as biomarkers for PCOS detection^101^, underscoring the role of sex steroid-metabolizing microbes in this disease. However, it remains unclear whether the enzymatic shift in the estrobolome precedes or follows the development of PCOS.

Testosterone also has substantial systemic effects: in healthy males^102^ and females^103^, exogenous testosterone administration increased muscle strength and altered body composition, while elevated levels in men have been linked to increased hemoglobin and hematocrit, reflecting erythropoietic effects^104^. Psychological effects include heightened aggression in males^105^ and shifts toward utilitarian decision-making in females^106^. Microbial sex steroid metabolism has been directly linked to these health outcomes: *Arthrobacter koreensis* (phylum Actinobacteriota), a testosterone-degrading bacterium isolated from the gut microbiota of male patients with depression^107^, reduced serum testosterone and induced depression-like behavior when introduced into mice, suggesting a role for microbial testosterone catabolism in mood regulation.

More speculatively, we propose that the biological activity of the testobolome—and its estrogen-metabolizing counterpart, the estrobolome—may be shifting under modern environmental pressures on the microbiome, including widespread antibiotic and medication use, and dietary changes^61^. Simultaneously, the incidence rates of several hormone-responsive cancers in young adults have risen, including testicular, endometrial, and estrogen receptor-positive breast cancers^108–110^. Similarly, ovarian and prostate cancer rates, which had been declining since early 20th century, now show signs of increase^108,109^. While the exact causes remain uncertain, changes in microbiome composition and its metabolic activity *vis-à-vis* sex steroids may be contributing factors. For example, obesity, a known modulator of the microbiome and vice versa, is a key predictor of increased estrogen levels^111,112^ particularly in postmenopausal women, and elevated estrogen burden has been associated with heightened cancer risk^113–118^.

## Future of the testobolome

The emerging concept of the testobolome—the collection of microbial enzymes and pathways that modulate testosterone metabolism and bioactivity—provides a new understanding about gut microbiota influences on host physiology. While the primary focus is on testosterone due to its abundance, centrality in steroid biosynthesis, and likely accessibility to microbes, downstream metabolites also merit attention. For example, we highlight the importance of investigating DHT, the most potent androgen responsible for systemic androgen-mediated effects^119^, as a potential target of microbial metabolism. Preliminary evidence implicates *Prevotella intermedius* (phylum Bacteroidota)^120^, a constituent of the oral microbiome, and fungi *Penicillium chrysogenum* and *P. crustosum*^121^, although a systematic classification of microbe-DHT interactions is lacking.

Although microbial β-glucuronidases and dehydrogenases have been implicated in testosterone metabolism, the enzymatic landscape of the testobolome remains largely uncharted. Expanding the catalogue of microbial hydroxysteroid dehydrogenases, including less-studied isoforms, such as 20α- and 20β-HSD, will clarify how microbial activity intersects with host steroidogenic pathways, influencing not only testosterone but also progesterone and glucocorticoid metabolism. Therefore, the systematic identification of microbial endocrinology^122^ should be a research priority, alongside the exploration of additional microbial processes, such as side chain cleavage and de novo synthesis of steroid-like metabolites.

Contributions from non-bacterial members of the microbiome, including fungal steroid-modifying enzymes, remain virtually unexplored but may add functional breadth to the testobolome^123^, considering that fungi engage in complex interactions with the host, bacteria, viruses, diet, disease, and the environment^124^, making the mycobiome an understudied target for testosterone metabolism research.

Resolving the quantitative and mechanistic basis of microbial testosterone metabolism will require the integration of stable-isotope tracing, strain-resolved metagenomics, and compartment-specific metabolomics to track sex steroid flux across the liver-gut-systemic axis. Such work should explicitly link microbial transformations to host conjugation pathways, transport systems, and binding proteins, capturing how these interactions alter circulating and tissue-level hormone pools. Context-specific variation influenced by sex, age, reproductive stage, adiposity, or environmental exposures must be incorporated into study designs to understand how the testobolome responds to perturbations, such as diet, antibiotics, and other pharmaceuticals.

Equally important is the development of robust analytical and computational infrastructure. Standardized metabolomic panels capable of resolving androgen conjugates, isomers, and epimers, coupled with curated databases of microbial steroid transformations, would accelerate reproducibility and cross-study comparisons. Advanced statistical and causal inference frameworks are essential to disentangle correlation from causation and to connect microbial taxa, genes, and metabolites to specific physiological outcomes.

Finally, translating testobolome research into clinical impact will require longitudinal human and model organism studies, linking microbial sex steroid metabolism to reproductive health, metabolic regulation, mood disorders, infectious disease susceptibility, and cancer risk. Therapeutic modulation of the testobolome through diet, targeted probiotics, enzyme inhibitors, or microbiome-directed drugs holds promise but demands rigorous safety evaluation. In line with this, the consequences of increased estrogen levels have already motivated research into aromatase inhibitors^125^, antiestrogens^126^, and selective estrogen-receptor modulators^127^ that could limit peripheral estrogen synthesis and inhibit estrogen receptor activation. Additionally, the microbial production of non-androgenic anabolic steroids, such as 5β-androstanes, may offer a route to developing safer anabolic agents for clinical use, reducing the adverse effects associated with conventional testosterone therapy^15,128^. In parallel, elucidating how microbial androgen metabolism influences pathogen virulence and antibiotic resistance will deepen our understanding of host-microbe conflict and could inform novel strategies for infectious disease management.

Defining the enzymatic repertoire of the testobolome and linking these processes to specific host outcomes will lay the groundwork for targeted, evidence-based interventions to modulate microbial testosterone metabolism with precision and safety.

## Supplementary information

Supplementary information

## Data Availability

No datasets were generated or analysed during the current study.
