# Supplementary material for: The testobolome in microbial testosterone metabolism and human health
Source: NPJ Biofilms Microbiomes. 2026 Jan 9;12:9. doi: 10.1038/s41522-025-00861-0 (PMC12789469; doi:10.1038/s41522-025-00861-0)
Supplement: Supplementary file 1 — Supplementary information [file 41522_2025_861_MOESM1_ESM.pdf]

**Supplementary Information**

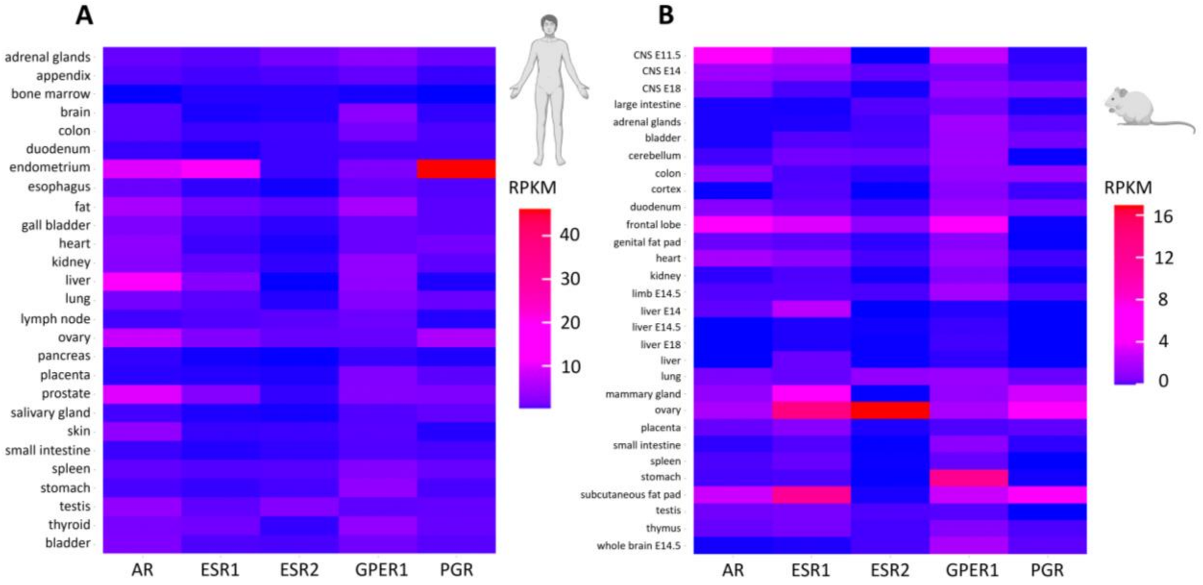


**Supplementary Figure 1. Preliminary assessment of tissue-specific expression of sex steroid receptors in humans and mice.** Heatmaps show the tissue-specific expression levels of androgen receptor (AR), estrogen receptors (ESR1, ESR2), G-protein-coupled estrogen receptor 1 (GPER1), and progesterone receptor (PGR) across human and mouse tissues. Expression is represented in RPKM (reads per kilobase of transcript, per million mapped reads), with higher values indicated in red and lower values in blue. (A) Human data (N=1–3/tissue) are from the Human Protein Atlas[1]. (B) Mouse data (N=1/tissue) are from the Mouse ENCODE Consortium[2]. Both datasets are publicly available through NCBI.


1. Fagerberg, L. *et al.* Analysis of the human tissue-specific expression by genome-wide integration of transcriptomics and antibody-based proteomics. *Mol. Cell. Proteomics* **13**, 397–406 (2014).
2. Yue, F. *et al.* A comparative encyclopedia of DNA elements in the mouse genome. *Nature* **515**, 355–364 (2014).
